# HLA-E Peptide Repertoire and Dimorphism—Centerpieces in the Adaptive NK Cell Puzzle?

**DOI:** 10.3389/fimmu.2018.02410

**Published:** 2018-10-17

**Authors:** Alexander Rölle, Dirk Jäger, Frank Momburg

**Affiliations:** ^1^Department of Medical Oncology, National Center for Tumor Diseasesm, University Hospital Heidelberg, Heidelberg, Germany; ^2^Clinical Cooperation Unit Applied Tumor Immunity (D120), German Cancer Research Center, Heidelberg, Germany; ^3^Antigen Presentation and T/NK Cell Activation Group (D121), German Cancer Research Center (DKFZ), Heidelberg, Germany

**Keywords:** adaptive NK cells, NKG2C, HLA-E, HLA-G, leader peptide, FcεRγ

## Abstract

Adaptive Natural Killer (NK) cells, a heterogenous subpopulation of human NK cells with a unique phenotypic and functional signature, became arguably one of the central areas of interest in the field. While their existence seems closely associated with prior exposure to human cytomegalovirus (HCMV), many questions regarding their origin and regulation remain unanswered. However, a common denominator for the majority of adaptive NK cells is the expression of the activating heterodimeric receptor CD94/NKG2C that binds to HLA-E, a non-classical HLA molecule, that displays a comparably restricted expression pattern, very limited polymorphism and presents a distinct set of peptides. Recent studies suggest that—in analogy to T cell responses—peptides presented on HLA-E could play an unexpectedly decisive role for the biology of adaptive NK cells. Here, we discuss how this perspective on the CD94/NKG2C-HLA-E axis aligns with the existing literature and speculate about possible translational implication.

The view of Natural Killer (NK) cells as exclusively innate lymphocytes has been challenged for more than a decade now. Ever since the seminal reports by Gumá et al. (1, 2), an increasingly clear picture of human NK cell subsets with adaptive features is emerging. Adaptive NK cells are characterized by a distinct epigenetic signature, alterations in key transcription factors, signaling adaptors, and cell surface receptors (3–6). This phenotype is accompanied by expansions of adaptive NK cell subsets resembling clonal T cell responses and a functional specialization that seems to favor antibody-triggered responses over natural cytotoxicity (7–9). These features are observed in different combinations on the single cell level, resulting in substantial heterogeneity within the adaptive NK cell subset. While we are far from understanding the biological relevance of this complex picture, two hallmarks—already defined in the early studies by López-Botet's group (2, 10)—remain unifying features: (1) The majority of adaptive NK cells expresses the activating heterodimeric lectin-like receptor CD94/NKG2C that binds the non-classical HLA-E molecule. (2) Prior exposure to human cytomegalovirus (HCMV) usually seems to precede the emergence of adaptive NK cells, even though adaptive NK subsets have been described in NKG2C-deficient individuals as well where CD2 engagement can compensate for the lack of NKG2C-mediated co-stimulation of antibody-driven responses (11). Other viral infections have been described to trigger the expansion of adaptive subsets, but it seems that this expansion does not take place in CMV-seronegative donors (10, 12, 13). Since primary CMV infection is usually asymptomatic, most reports are likely studying secondary expansions that accompany CMV reactivation events, e.g., after transplantations (14–16). The molecular events underlying the initial formation of the adaptive NK cell subset remain strikingly enigmatic.

Very recently, Hammer et al. (17) as well as our own group (18) highlighted the critical importance of peptides, most notably a peptide derived from the leader sequence of HLA-G, presented on HLA-E for the regulation of adaptive NK cells, further emphasizing the role of this receptor-ligand system. We speculate here that the remarkable degree of peptide specificity together with certain features of the HLA-E biology make a compelling case for the HLA-E/NKG2C axis as the central driver for CMV-induced generation of adaptive NK cells.

We will set out by recapitulating some evolutionary aspects of HLA-E molecules that need to be considered with regard to the ongoing battle between the mammalian immune system and persistently infecting viruses, which holds in particular for the beta-herpes virus CMV that has developed a multitude of immune escape strategies targeting innate as well as adaptive immune responses.

The non-classical MHC class I molecule HLA-E plays a threefold role in the regulation of certain aspects of the innate and adaptive immune system. Firstly, HLA-E molecules loaded with peptides derived from various HLA class I ER leader sequences block conventional NK cells expressing the inhibitory heterodimeric NKG2A/CD94 receptor containing two ITIM motifs in the cytoplasmic domain of NKG2A (19–25). Secondly, HLA-E molecules preferentially loaded with the HLA-G leader peptide have the capacity to trigger ADCC-competent “adaptive” NK cells expressing the activating NKG2C/CD94 receptor that is associated with the ITAM-containing adaptor molecule DAP12 (17, 18, 22, 26, 27). Thirdly, HLA-E molecules presenting various self and virus-derived peptides can be ligands for CD8^+^ cytotoxic T cells expressing αβ T cell receptors (28–33). HLA-E-restricted T cell responses are, however, beyond the focus of this *Perspective*.

## Unique evolutionary features of dimorphic HLA-E molecules

Evolutionary studies indicate that the HLA-E locus is the most well conserved among all primate MHC major histocompatibility complex (MHC) class I genes (34) indicating that it serves specialized functions in the immune system distinct from the highly polymorphic HLA-A, B, and C locus products. In humans two allelic variants can be distinguished in Caucasian populations that differ only in amino acid 107 (Arg in HLA-E^*^0101, Gly in HLA-E^*^01031 and E^*^01032) (35, 36). Amino acid 107 is located in an outwardly exposed loop below the α_2_-helix of the peptide-binding platform and does not affect the general structure of peptide-assembled HLA-E molecules (37). While HLA-E^G^ seems to be older allele since Gly107 is exclusively found in all primate HLA-E orthologs, population studies suggest that the HLA-E^R^ single nucleotide polymorphism has emerged before the expansion of *Homo sapiens* (38). HLA-E^G^ and HLA-E^R^ alleles occur in about equal frequencies in different ethnic groups and are maintained in diverse ancestral HLA haplotypes by stabilizing selection (38). While influences of the genetic HLA-E dimorphism on graft-vs.-leukemia reactions after hematopoietic stem cell transplantation, spontaneous abortions, viral infections, and susceptibility to autoimmune diseases have been described elsewhere (39–42), we will focus here on features of HLA-E proteins related to the formation of ligands for CD94/NKG2A/C NK receptors.

## Peptide-loaded HLA-E molecules as binding partners for NKG2A/C

While HLA-E transcripts show a broad tissue distribution (43), surface expression of of HLA-E proteins is mainly restricted to resting and activated T cells, NK cells, B cells, monocytes, and macrophages as well as endothelial cells (23, 44). Hence NKG2A-expressing NK cells that circulate through blood vessels and lymphoid tissues will constantly be exposed to varying levels of inhibitory stimuli. Due to the ~6-fold lower affinity of peptide-loaded HLA-E molecules to NKG2C (45, 46) and stricter peptide selectivity of the HLA-E/NKG2C interaction (17, 18, 22, 47) it seems, however, more unlikely that NKG2C^+^ NK cells will receive tonic stimulation under physiological conditions. While HLA-E was noted to possess generally low surface expression levels as compared with HLA-A and B molecules, the HLA-E^G^ allotype loaded with different peptides shows consistently higher surface expression than HLA-E^R^ (37, 48, 49). This can be attributed to various factors including less efficient assembly with β_2_-microglobulin and slower ER egress, lower affinity for all tested HLA leader peptide ligands and reduced thermostability of the HLA-E^R^ variant (37, 48, 49). This suggests that background NKG2A/C engagement will be very low in the HLA-E^R^ homozygous situation which might reduce the inhibition/activation threshold of NKG2A^+^/C^+^ NK cells, but also of NKG2A^+^ T cells, during viral infection and other pathological conditions (50). In this context it is interesting to note that the presence of the HLA-E^G^ variant was reported to be associated with higher incidence of CMV infection after kidney transplantation (51), which might be related to a more pronounced dampening of NKG2A^+^ NK cell responses.

The HLA-E ligands for NKG2 family members are usually formed after loading HLA-E molecules with 9-mer peptides processed out of ER leader sequences from various HLA-A, B, and C allotypes as well as HLA-G in a TAP- and proteasome-dependent fashion (22, 24, 25, 52–54). HLA-E-stabilizing leader peptides that confer protection from NK cell lysis by binding to NKG2A have the consensus sequence VM(A/P)PRT(L/V) (V/L/I/F)L and thus exclude several HLA-B allotypes (containing a Thr or Ala residue instead of Met), a few HLA-C allotypes and the leader peptides from HLA-F and HLA-E itself that do not match this motif. HLA-E molecules thereby monitor the biosynthesis of most polymorphic class I allotypes as well as the class Ib molecule HLA-G and regulates NK cell activity as a functional complement to the polymorphic KIR system.

During cellular stress Hsp60 is upregulated and can give rise to a competing HLA-E ligand (55). HLA-E/Hsp60 leader peptide complexes are *not* bound by NKG2A/CD94 and thus provide a mechanism for NK cells to specifically attack stressed cells (55). In addition to the Hsp60 peptide, a great number of non-canonical, sometimes pathogen-derived HLA-E ligands (with striking differences between HLA-E^G^ and HLA-E^R^) have been identified (56–59) that will probably be of little relevance for NK cell recognition.

By clear contrast, the requirements for the recognition of peptide-loaded HLA-E molecules by NKG2C/CD94 are much more restricted. It was noted that the HLA-G-derived leader peptide VMAPRTL**F**L in complex with HLA-E has a dominant role in inducing cytotoxic activity in NKG2C^+^ NK cell clones using peptide-pulsed, HLA-E^*^0101-expressing 721.221 B-lymphoblastoid cells or PBMC as stimulators (22, 47). Using microspheres charged with recombinant peptide-loaded HLA-E^*^0103 molecules we have recently shown that only the HLA-E^pHLA−G^ complex is able to trigger FcεRIγ downmodulation, IFN-γ release, CD25 upregulation, proliferation, and ADCC responses in NKG2C^+^ NK cells (18). The pivotal role of the HLA-G peptide for NKG2C/CD94 stimulation *in vitro* appears to be in accordance with biochemical studies analyzing the affinities and thermodynamic parameters of NKG2x/CD94–pHLA-E interactions (46). Crystal structures surprisingly revealed that the critical Phe_8_ residue in the HLA-G peptide is in contact with CD94 but not with the differentially regulated NKG2A/C chains (60, 61). The predominance of the HLA-G peptide-loaded HLA-E for adaptive NK cells prompts questions regarding the natural availability of such complexes in light of the restricted tissue distribution of HLA-G (62–64).

## Human cytomegalovirus (CMV) influences the HLA-E/NKG2 interaction

Human cytomegalovirus has highjacked the HLA-E/NKG2A axis for the purpose of immune evasion. In the presence of TAP peptide transporter blockade through the CMV protein US6, HLA signal peptide-mimicking sequences derived from the CMV glycoprotein UL40 are able to supply HLA-E molecules with stabilizing peptides TAP-independently (65, 66). Interestingly, the early HCMV gene products US2 and US11, that attack HLA-A, B, C, and G molecules in an allotype-specific fashion, do not downmodulate HLA-E heavy chains (67); and the HLA-E^G^ but not the HLA-E^R^ alloform may be resistant to TAP blockade by US6 (67, 68). The HCMV glycoprotein US10 selectively targets the NK-inhibitory HLA-G molecule for degradation while HLA-E levels are only slightly affected (69). Since HLA-G is inserted into the ER membrane before US10-mediated degradation, HLA-G leader peptides will likely remain available for HLA-E loading and NKG2C interaction in CMV-infected tissues unless their ER entry is blocked by the late HCMV gene product US6. We conclude that HLA-E appears to be remarkably spared from the attack of HCMV immunoevasins principally favoring not only the blockade of conventional NKG2A^+^ NK cells but simultaneously also the expansion of NKG2C^+^ NK cells.

## Adaptive NK cells benefit from HLA-E loaded with CMV UL40-derived peptides

There seems to be an ongoing coevolution likely resulting in mutual benefit for the propagation of HCMV variants and the persistently infected human host. This assumption is underpinned by the finding that in clinical HCMV isolates, the UL40 protein shows a mutational hotspot at position 8 of the potential HLA-E-binding peptide at position UL40(15–24) (70). Some UL40 variant peptides reduced the affinity of the interaction between HLA-E and CD94/NKG2A and some selectively reduced the NK cell-mediated lysis by NKG2C^+^ NK cell clones (70). Notably, UL40 is endowed with a dual function and does not only provide HLA-E binding sequences but also promotes UL18 expression (71). The UL18 gene product is a high affinity decoy-ligand for the inhibitory receptor LIR-1 (72), that is highly expressed on adaptive NK cells and that mediates decreased susceptibility of HCMV-infected cells to LIR-1^+^ NK cells (73). This could suggest that the immune-evasive functions of UL40 are primarily targeted at adaptive NK cell populations. In a very profound analysis it was recently shown that UL40-encoded peptides control the activation, expansion and differentiation of NKG2C^+^ adaptive NK cells in a subtle fashion (17). A rare UL40 peptide identical with the above-mentioned HLA-G leader peptide was confirmed to be optimally stimulating, followed by a frequent UL40-derived peptide mimicking the leader peptide present in most HLA-C alleles (VMAPRTL**I**L) (17). This study elegantly recapitulated prior knowledge regarding the epigenetic imprinting and expansion of adaptive NKG2C^+^ NK cells in the presence of genetically diverse HCMV strains. The authors propose that different strain-specific UL40-derived peptides account for the heterogeneity of adaptive NK cell populations.

## HCMV-driven priming and expansion of adaptive NK cells

The relevance of HLA-E-NKG2C interactions for the expansion of adaptive NK cells has been demonstrated *in vitro* (2, 74, 75, 76) and is supported by studies showing that NKG2C zygosity directly correlates with NKG2C surface levels and the size and distribution of NKG2C^+^ NK cells *in vivo* (77–79).

Interestingly, Gumá et al. had already studied the influence of HLA-E alleles—and thereby presumably HLA-E surface levels—on HCMV serology and percentage of NKG2C^+^ cells. While the limited number of subjects makes definitive conclusions challenging, the authors noted a weaker correlation between NKG2C expression and HCMV serology in individuals with the HLA-E^G^ allele, i.e., potentially higher HLA-E expression (1). Does this indicate a more successful antiviral NK cell response via NKG2C–HLA-E^G^ which is in turn reflected by reduced antibody responses? Or is an increased ligand engagement of NKG2C, both under homeostatic conditions as well as during HCMV infection, an underlying reason for the lower percentage of cells with the NKG2C receptor in HLA-E^G^ homozygous individuals?

An intriguing open question remains why HCMV appears to have such a decisive role for the generation and expansion of adaptive NK cell populations (80). What is the difference compared to other pathogens, and which combination of parameters is unique to cytomegalovirus? Together with recent studies, that show the profound impact of the HLA-G-derived leader peptide (17, 18), we are tempted to speculate that only CMV infection provides both, elevated HLA-E and HLA-G expression on a single cell level. Interestingly, a polymorphism in the 3′-untranslated region of the HLA-G gene that affects HLA-G expression levels has been reported to modify susceptibility to CMV infection (81). Macrophages and monocytes express HLA-E (23, 44) and *de novo* induction of monocyte HLA-G cell surface expression was reported for active HCMV infection (77) and reactivation of latent infection *in vitro* (82). HLA-G upregulation in monocytes/macrophages is supported by increased levels of cellular IL-10 and cmvIL-10 (HCMV gene product UL111A) during infection while expression of classical MHC class I molecules is suppressed (83, 84). Intriguingly, monocytes, monocyte-derived macrophages, and DCs were identified as the major cellular target for cmvIL-10 and hIL-10 (85). Co-expression of HCMV gene products and HLA-G has been demonstrated for macrophages with reactivated CMV infection on a single-cell level (82). In line with studies using cell lines overexpressing CMV immunoevasins US2, US3, or US6 (86, 87), other researchers noted, however, a down-regulation of HLA-G cell surface expression in freshly CMV-infected astrocytoma cells *in vitro* (88). For a successful loading of HLA-E molecules with HLA-G leader peptide the activity of the TAP-blocking HCMV immunoevasin US6 is, in our opinion, of foremost relevance. US6 expression levels peak only around 48 h post-infection and then slowly decrease (89). Since US2 and US11, showing maximum expression levels within 24 h post-infection (89), do not affect HLA-E surface expression (67) we propose that there is a sufficient time window for the formation of HLA-E^pHLA−G^ complexes in a subset of acutely CMV-infected monocytes/macrophages before TAP inhibition through US6 sets in and HLA-E continues to be supplied with UL40-derived TAP-independent peptide ligands. The study by Onno et al. (82) indicates that even 20 days after reactivation of latent CMV high HLA-G levels can be observed in infected macrophages while only a partial down-modulation of HLA-A,B,C molecules was noted, suggesting that the activity of US6 may have ceased. In this setting, which may aptly reflect the *in vivo* situation of a chronic CMV infection with sporadic reactivations, HLA-E^pHLA−G^ complexes should be abundant.

A similar setting seems possible for endothelial cells that were reported to trigger NKG2C^+^ NK cells upon primary CMV infection (90) and that represent a major non-lymphoid cell type displaying substantial HLA-E expression, particularly under inflammatory conditions (44). This would permit a scenario of sufficient HLA-E complexes, presenting the VMAPRTLFL peptide. Since both, monocytes as well as endothelial cells are considered reservoirs of viral latency (91), they could also act as critical mediators for secondary expansion of adaptive NK cells upon CMV reactivation which aligns with *in vitro* data (6, 75, 90) and a study that observed a correlation between monocyte counts and the magnitude of NKG2C^+^ NK cell expansion during reactivation (92).

If we consider the differential reactivity of NKG2C with HLA-E-bound leader peptides together with the allelic dimorphism of HLA-E itself, it becomes clear that one has to envisage a range of NKG2C ligands with different affinities expressed at different surface densities. Weaker NKG2C ligands were shown to require costimulation through CD2 for adaptive NK cell triggering through CD16 and to have a lesser impact on gene methylation (17). The VMAPRTLFL peptide is set apart from other HLA-E ligands by its strong impact on adaptive NK cell populations. Whether it is primarily derived from rare UL40 variants as described by Hammer et al. (17) or more often the result of increased cellular HLA-G levels as a consequence of HCMV infection remains an open question.

**Figure 1:**
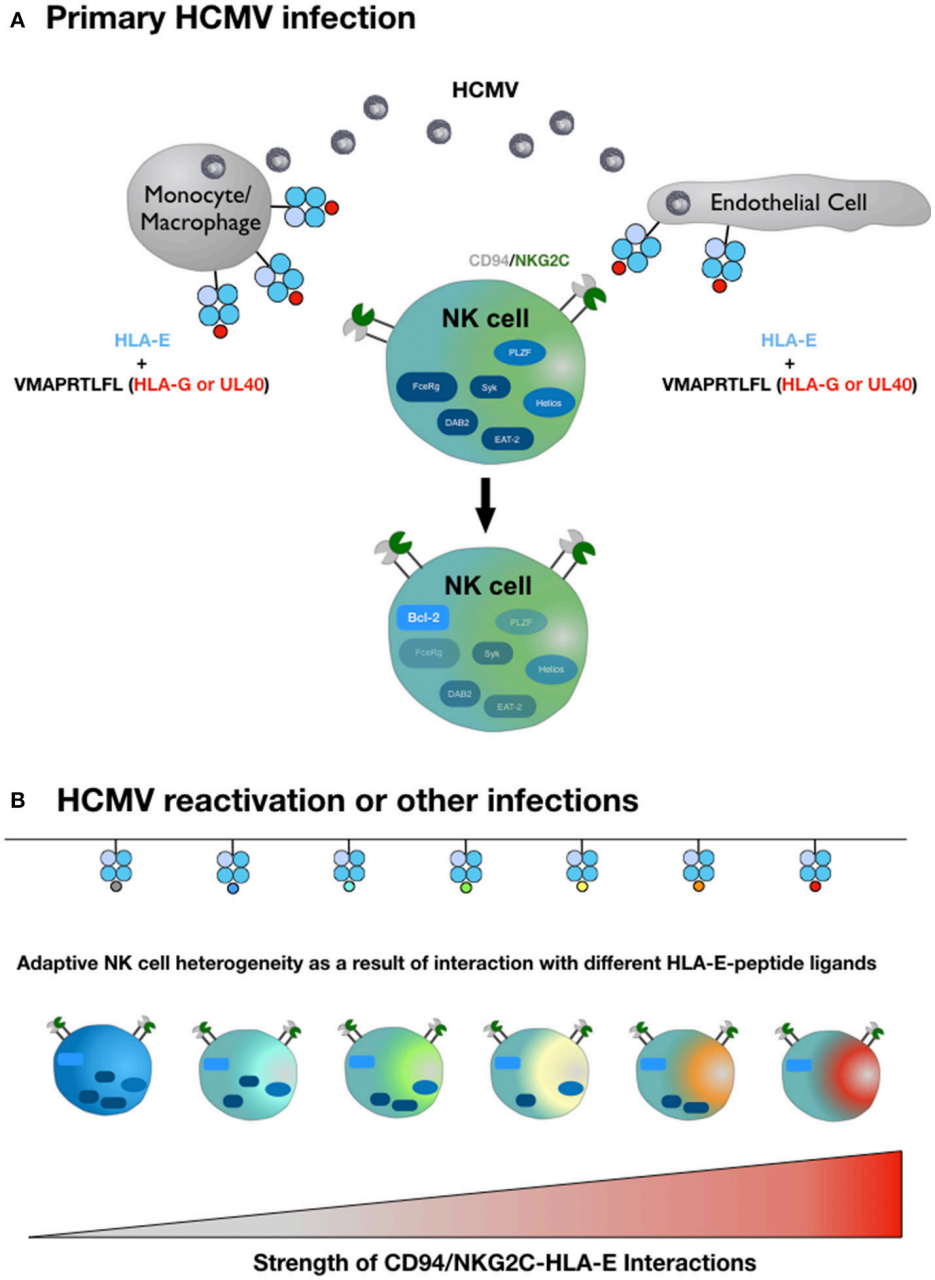
Hypothetical model of adaptive NK cell differentiation driven by the HLA-E ligandome. **(A)** Monocytes/macrophages and endothelial cells represent the primary site of CMV infection and consecutive latency. Simultaneous expression of HLA-E and increased availability of HLA-G-derived peptides—either derived from rare viral UL40 variants or from cellular HLA-G, upregulated during infection—generate HLA-E molecules in complex with the HLA-G-derived peptide VMAPRTLFL. This strong ligand triggers adaptive NK cells (or precursors) via the heterodimeric CD94-NKG2C receptor, which initiates epigenetic imprinting and the gradual loss of signaling molecules, e.g., FcεRγ or transcription factors, e.g., PLZF. **(B)** Reactivation of CMV from latency reservoirs or other infections that affect HLA-E levels and/or the pool of presented peptides provide a diverse range of ligands for CD94-NKG2C. Depending on the respective affinities and the ensuing interactions, adaptive NK cells continue to downregulate additional molecules of intracellular signaling, eventually giving rise to an increasingly heterogenous population of adaptive NK cells. For reasons of clarity, the figure does not take into account that different HLA-E alleles result in different HLA-E expression levels and that infections are accompanied by distinct cytokine signatures. We also omit the characteristic modulation of cell surface receptors on adaptive NK cells compared to canonical NK cells and the potential need for costimulation for weak HLA-E-peptide ligands.

In light of the exquisite peptide specificity of adaptive NK cells, we hypothesize that the strength of the HLA-E/NKG2C interaction in conjunction with variable conditions of costimulation and a favorable cytokine milieu is the molecular basis for the complex inter- and intraindividual heterogeneity of adaptive NK cells (Figure 1). To test this hypothesis, future studies have to systematically dissect the impact of different HLA-E-peptide ligands on the characteristic epigenetic profile and altered intracellular signaling of adaptive NK cells.

## Adaptive NK cells in cellular immunotherapy—a viable option for HLA-E^+^ tumors?

Mounting evidence indicates that CMV reactivation can result in a reduced relapse rate in acute myelogenous leukemia (93, 94) and chronic myelogenous leukemia (95). Cichocki et al. observed that patients with hematologic malignancies receiving HSCT displayed a selective and sustained expansion of adaptive NK cells after experiencing CMV reactivation (92), and another report showed higher cytotoxicity of adaptive NK cells from CMV-seropositive donors compared to seronegative donors (96). This suggests that adaptive NK cell subsets can mediate a substantial anti-leukemia effect, even though the molecular mechanisms remain unclear at this point. A recent study demonstrated that the reactivity against primary pediatric ALL blasts is largely independent of CD94(/NKG2C)-HLA-E interactions but rather relies on “missing-self” recognition (97) and possibly DNAM-1 (98). However, NK cell activation and receptor usage can vary dramatically depending on the cellular context and the therapeutic potential of adaptive NK cells, particularly in tumor entities that are characterized by increased HLA-E levels, merits further investigation. High expression of non-classical MHC class I molecules is often associated with poor clinical prognosis (62, 64, 99), rendering those malignancies a potential scenario for the utilization of NKG2C^+^ NK cells. Cellular immunotherapies might also benefit from other favorable properties, e.g., elevated resistance to MDSC and T_reg_ suppression, that were ascribed to adaptive NK cell populations (100, 101). Given their superior capacity for mediating ADCC, adaptive NK cells could also be combined with therapeutic interventions relying on monoclonal antibodies, bi-specific or tri-specific killer engagers (BiKEs/TriKEs) (102).

First steps toward devising clinical protocols for the use of adaptive NK cells have been taken and promise cellular NK cell-based therapies with higher efficiency (97). Expansion of NKG2C^+^ cells by HLA-E expressing transfectants is a successful strategy for obtaining robust proliferation (74, 97) of functional adaptive NK cell populations. In light of recent findings regarding the remarkable peptide specificity of adaptive NK cells (17, 18, 47) a further optimization of these approaches by tailoring the peptide ligand on HLA-E appears desirable.

## Author contributions

AR defined the scope, wrote the manuscript, and generated the figure. DJ discussed the underlying concepts. FM discussed the underlying concepts and wrote the manuscript.

### Conflict of interest statement

The authors declare that the research was conducted in the absence of any commercial or financial relationships that could be construed as a potential conflict of interest.
